# New Challenges with Treatment Advances in Newborn Infants with Genetic Disorders and Severe Congenital Malformations

**DOI:** 10.3390/children9020236

**Published:** 2022-02-10

**Authors:** Rahel Schuler, Ivonne Bedei, Frank Oehmke, Klaus-Peter Zimmer, Harald Ehrhardt

**Affiliations:** 1 Department of General Pediatrics and Neonatology, Justus Liebig University, Feulgenstrasse 12, D-35392 Giessen, Germany; klaus-peter.zimmer@paediat.med.uni-giessen.de (K.-P.Z.); harald.ehrhardt@paediat.med.uni-giessen.de (H.E.); 2 Department of Obstetrics and Gynecology, Justus Liebig University, Klinikstrasse 33, D-35392 Giessen, Germany; ivonne.bedei@gyn.med.uni-giessen.de (I.B.); frank.oehmke@gyn.med.uni-giessen.de (F.O.)

**Keywords:** genetic disorders, severe congenital malformations, prenatal counselling, treatment options, quality of life

## Abstract

Advances in the prognosis of relevant syndromes and severe congenital malformations in infants during the last few decades have enabled the treatment and survival of an ever-increasing number of infants, whose prospects were previously judged futile by professional health care teams. This required detailed counselling for families, which frequently started before birth when a diagnosis was made using genetic testing or ultrasound. Predictions of the estimated prognosis, and frequently the more-or-less broad range of prospects, needed to include the chances of survival and data on acute and long-term morbidities. However, in the interest of a having an informed basis for parental decision-making with a professional interdisciplinary team, this process needs to acknowledge the rights of the parents for a comprehensive presentation of the expected quality of life of their child, the potential consequences for family life, and the couple’s own relationship. Besides expert advice, professional psychological and familial support is needed as a basis for a well-founded decision regarding the best treatment options for the child. It needs to be acknowledged by the professional team that the parental estimate of a “good outcome” or quality of life does not necessarily reflect the attitudes and recommendations of the professional team. Building a mutually trusting relationship is essential to avoid decision conflicts.

## 1. Introduction

Within the last few years, there has been substantial progress in in utero diagnostics. There is a wide spectrum of conditions, ranging from genetic syndromes with differing prognoses to malformations of varying severities, that are diagnosed prenatally. This leaves parents and clinical team with the difficult decision of whether a pregnancy should be continued or terminated, and how the child would be treated postnatally. Treatment options range from an interventionist approach, including operations, to a palliative care approach, also described as comfort care.

Survival rates, disability free survival, length of hospital stay, required operations, need for intensive care, etc., are important aspects that are generally discussed during the pre- and postnatal counseling of parents.

Although important, it is often challenging to provide parents with accurate data. Case numbers are frequently low, the range of outcome wide, and estimated outcomes can depend, e.g., on additional malformations that are detected prenatally (or not) [1]. This is in contrast to the frequent situation of premature births or classical diseases in childhood and adulthood, where large cohort registries or prospective observational studies guide treatment advances and lifetime perspectives.

## 2. Framework for Counseling of Parents

### 2.1. Survival

For many congenital diseases, survival has improved substantially due to medical progress [2,3,4], but still continues to be much lower than in the reference population [4]. Survival is obviously greatly dependent on the type of congenital anomaly, e.g., children born in recent years with Down syndrome (DS) have a life expectancy of 60 years [5] and 1-year survival is above 90%; for those born with congenital diaphragmatic hernia (CDH), survival is greater than 80%; and 1-year survival is only 15% for children with trisomy 18. Even for children with the same congenital anomaly, survival differs and depends on the presence of additional malformations, low birthweights, preterm births [4] and, where applicable, on the provision of perinatal interventions [6]. In Japan, for example, a more interventionist approach for trisomy 18 is common. A significantly increased 28-day survival of 83% has been documented using this approach, as opposed to 37% in a large population-based study from the United States [7,8]. In a single-center evaluation in the United States survival was improved with a non-palliative approach, although there was no difference in survival whether more or less aggressive interventions were performed [6]. Considering these data, the continuing practice of referring to trisomy 13 or 18 as “lethal” or “incompatible with life” is clearly not warranted [9]. Survival of patients after the Fontan procedure and after surgical repair of a CDH has significantly increased over the last few decades [3,4].

### 2.2. Morbidities

Despite medical progress, most children with genetic syndromes or severe congenital malformations have long-term morbidities and medical treatment is required for several years or is even life-long [3].

Children with trisomy 13 or 18 who survive the neonatal period all have significant neurodevelopmental impairment, but many of them are able to reach some milestones, e.g., smiling, playing with toys, or eating using their mouth [10]. After the Fontan procedure, long-term morbidity, re-operations, and re-interventions remain common [3], survivors after CDH repair may have neurodevelopmental impairment, long-term lung disease, and gastrointestinal morbidities [3,11,12].

During pre- and postnatal counseling, the focus often is on these two outcome parameters. What is less considered, but is at least equally important as survival and morbidities for the individual and the whole family is the quality of life (QoL) or health-related quality of life (HRQoL) up to adolescence or adulthood, the impact a disabled child has on family life, siblings, and marriage, and the view of other families in similar situations. Of importance, for most conditions with an uncertain outcome, expectations for long-term quality of life (or for living an independent life) are even harder to outline while these items are available, for example, for prematurely born infants.

### 2.3. Quality of Life

Several studies assessing HRQoL through parent questionnaires found the HRQoL of children, adolescents, and adults with DS to be lower than the HRQoL of people without DS [13,14,15,16]. Interestingly the self-reported QoL of people with DS is much better. The overwhelming majority of people with DS rate their own lives as happy and fulfilling. A total of 99% of respondents to a survey in the USA of people with DS aged 12 years and older answer the question: “Are you happy with your life” with “Yes”, 97% of respondents like who they, are and only 4% were sad about their life [17]. The same survey in Japan yielded similar results [18].

The difference between proxy- and self-reported QoL is a common finding. A comparison of parent versus child reported that the functional health status of children after the Fontan procedure showed that the child had a better perception of their own health, a higher self-esteem, and a better mental health in comparison to the parental perception [19]. The same is true for previous preterm infants [20], whereas the opposite was found in a study assessing the HRQoL of survivors of CDH: the parent reported HRQoL was better than the child report [21]. Generally, the QoL and HRQoL of children with CDH appears to be the same as in the healthy population, without a significant association between prenatal severity of disease and QoL, but lower for those more affected postnatally and still in need of support [12,22,23].

Data are scarce in this field regarding self-reported QoL in infants with genetic syndromes and severe congenital malformations and more research is desperately needed, as clearly proxy-reports cannot replace self-reported QoL. There are genetic syndromes and diseases with severe neurological impairment, making self-report impossible, but, even in these cases, one has to keep in mind that the proxy-report does not reveal the full picture. Whenever possible, one should strive for self-reports and adapt tools accordingly. In the absence of self-reported QoL data, parents seem to be a better proxy than healthcare professionals (HCPs), as there is more consistency in rating the HRQoL of more severely disabled health statuses between adolescents and parents than between HCP and adolescents or HCP and parents [24]. Another limitation one has to keep in mind regarding assessment of QoL is that it involves young adults and, therefore, assesses those who received a treatment that was common 10–20 years earlier. With new options regarding treatment strategies, including operations and medical follow-up programs, as well as psychosocial support, QoL may be different for patients whose parents receive counselling today.

### 2.4. Family Life

The impact of a child with a disability on the family is significant. Often, having a child with a disability involves prolonged hospital stays, operations, doctors’ visits, and, for some, it even means early death. Therefore, commonly, during prenatal counselling, there is a focus on the negative impact these children have on families, siblings, parents, and the financial situation [10]. In surveys of parents of a child with DS or trisomy 13 or 18, this view could not be confirmed [10,25]. Parents of children with DS described significant learning difficulties, struggles, and challenges, but the majority of parents experienced enrichment and a more positive outlook on life and reported a good relationship with siblings [25,26]. Although parents of children with trisomy 13 or 18 described financial strain, challenges of caring for a child with special needs, and acknowledged that the child had more pain than other children, the overwhelming majority described their child as happy (99%) and felt that their child enriches or enriched their life (98%). Of those with other children, 82% described a positive effect on the siblings [10].

In the absence of national registries for people with DS, these surveys were conducted with parents that are part of patient organizations or members of social networks; therefore, these are not representative of the whole population with DS or trisomy 13 and 18 [25,27]. Despite these limitations, the sample size was large and these views of parents need to be integrated into prenatal counselling [28].

Families pursuing treatment for their child with hypoplastic left heart syndrome describe different experiences from those with genetic syndromes. They did report of a better family functioning than published norms from 2 to 16 months after birth, but parents had a significantly lower QoL and well-being [29]. Parents of children with CDH had higher parenting stress levels, especially those whose children had a more severe course with longer hospital stays and the requirement of ECMO support [30].

### 2.5. Marriage

A common concern within medical teams is that the birth of a disabled child has a negative impact on marriage and increases divorce rates. Evidence for this is lacking.

Although 11% of parents of children with DS did report a strain on their marriage [25] in a large population-based study, parents of children with DS had a lower divorce rate (7.6%) than parents without a disabled child (10.8%). Parents of children with other birth defects had a divorce rate of 11.2%, which was also not relevantly higher than those in the comparison group [31]. A Brazilian survey confirmed this finding for parents of children with DS [32]. Parents of children with trisomy 13 or 18 reported a divorce rate of only 3%, whereas 68% reported positive effects on their relationship [10]. In a Turkish study, the divorce rate of parents with a disabled child were low and most reported positive effects on their relationships [33]. Therefore, the available data do not support the idea that having a disabled child increases divorce rates.

### 2.6. Experience Exchange with Other Affected Families

The parents of children with genetic syndromes appreciate being informed about the views and experiences of other parents who had or have a child with the same genetic syndrome or malformation, or to be connected with individual parents or support groups [10]. When asked about their advice to expectant couples, many parents of children with DS would describe the joys and rewards of raising a child with DS, they would also be honest about the struggles and challenges and would encourage them that “what at first appears to be the worst possible thing that could be happening, can turn into the best possible thing” [25]. Overall, parents of children with trisomy 13 or 18 have a very positive perspective on the impact their children have or had on the family, and described their children’s lives as valuable and purposeful [10,34]. While parents did describe financial challenges, the majority described their family as being strengthened; 98% reported that their child has enriched their lives [10].

Parents of children with hypoplastic left heart syndrome described financial difficulties, experiences of stress and anxiety, but also of gratitude for each day they had with their child. The majority of parents were doing well and reported that the best coping strategy was to treat the child as a normal part of the family [35].

### 2.7. Prenatal and Postnatal Attachment

Unfortunately, there are no data on prenatal and postnatal attachments of parents to their disabled or severely ill child, and how this will influence the infant and the whole family later in life. Data regarding differences in the impact on family life and marriage, depending on whether the diagnosis was made pre- or postnatally, are also lacking and are interesting areas of research for the future, e.g., DS.

## 3. Determining the “Best” Treatment Options

To determine which treatment is suitable for each individual child, the health care practitioner (HCP) needs to see the individual patient with their unique set of malformations and not a syndrome. Others have also suggested this more personalized approach, which focuses more on the uniqueness of the individual than on the population average when deciding treatment options [36]. It is helpful to understand parents’ perspectives on what a “good outcome” for their child would be. To enable parents to define this “good outcome” for their individual child during pre- and postnatal counselling, it is important to provide whatever evidence exists on mortality, survival, expected neurodevelopment, longer-term morbidities, QoL, and views from other parents. At times, this is difficult, sometimes even impossible, as some of these syndromes are rare with a wide spectrum of associated malformations and, therefore, wide variations in outcome. Discussing these limitations in outcome prediction should also be part of these consultations.

For some genetic syndromes and congenital malformations, up to 80% of parents decide for termination of pregnancy [37]. While some make this decision after prenatal counselling, including all different treatment options, others do not receive such comprehensive information. A significant number of parents of children with trisomy 13 or 18 experienced the pressure to terminate the pregnancy and felt being judged if deciding for more than comfort care [28].

Therefore, it is important to discuss all treatment options with expectant parents. These include palliative care, also called comfort care, without any medical interventions on one side of the spectrum and full provision of interventions, including resuscitation, mechanic ventilation, and surgeries on the other. Between these two extremes, there are many possible individual approaches. To work with parents to determine what they wish for their individual child is the responsibility of the medical team based on legal aspects, guidelines, recommendations, and published outcome results. This usually requires a decision process and parental psychological and familial support. Defining what the “good outcome” for a child is can be helpful in this way [38]. For some, the “good outcome” might be unimpaired survival, but the majority of parents report a “good outcome” to be any survival, the possibility to take the child home, being able to hold the child alive, having tried and given the child the opportunity to survive [1,28,34]. Obviously, the definition of this “good outcome” will differ between families due to different spiritual backgrounds, cultures, and values.

In some situations, clinicians might feel that the treatment parents want for their child is not in the best interest of the child and find it difficult to continue or start interventions they feel are futile. HCPs might feel that a short life and a good death are in the best interest of the child [28]. Instead of labelling a medical intervention as “futile” if death cannot be averted, medical interventions could be judged as futile or not with regard as to whether the family goal of the “good outcome” is reached [28]. With this approach, surgery even for life-limiting syndromes like trisomy 13 or 18 might be an option for some families if it enables them to reach their goal of spending more quality time with their child and being able to take their child home. For families of these children the focus is on a good life even though it may be short instead of on a life as short as possible and a good death [28]. The challenge of physicians and parents alike is the balance between doing “too little” and risking early death or doing “too much” and letting the child suffer undue pain. Interestingly, when looking back, only 1% of parents with children with trisomy 13 or 18 felt they had agreed to too many medical interventions, while 24% felt they didn’t do enough [28]. Parents and former preterm infants, now adolescents, agree more on their judgement of the value of a child with severe disability than with HCPs [24]. Therefore, parents appear to be the better decision-makers who have the best interests of their severely disabled child in mind. The caring relationship of parent and child has been described as morally meaningful, even if the child has limited or no observable ability to reciprocate. Parents realize the relational potential even if the child is significantly intellectually disabled and completely dependent. It has been suggested that this inherent value of caring relationships is a strong reason for clinicians to provide life-sustaining treatment to a severely disabled child [39].

Differences regarding treatment intensity do not only exist between HCPs and parents, but also exist within clinical teams [1,40]. Naturally, within these teams, differences in personal beliefs, background, and values exist and influence what the individual clinician views as the “good outcome”. If different views within a professional team exist, and a consensus cannot be reached, it is advisable to counsel parents together and be open about the different possible approaches. While one has to acknowledge that pure fact based, value-free consultation might not be possible [38], it has to be self-evident that each child and their family deserves to be treated with respect and dignity due to their inherent value, irrespective of how fragile or disabled a child might be. Discontinuing the use of false statements like “lethal condition”, “incompatible with life”, “the child will ruin your marriage”, or “this child will live a meaningless life” is one step in the right direction [10].

Although, in general, parents are the best surrogate decision makers for their infants, there might be cases in which parents refuse consent to a treatment that HCPs assess as necessary and in the best interest of the child [41]. One such example is the refusal of parents for consent of surgery for duodenal atresia of an infant with DS [42]. The limit to parental decision-making authority has been suggested to be where the outcome of a treatment is clearly beneficial with a low likelihood of severe neurodevelopmental impairment. If no consensus can be reached during consultations with parents, it might be necessary to apply to the court system; this is certainly the least desirable option and can be avoided in most cases if HCPs succeed in building a trusting relationship with parents [41].

## 4. Conclusions

During pre- and postnatal consultations, it is important to provide parents with the best evidence there is on the expected outcomes of their child and the impact on the family, while also being open about the limitations all predictions have. All different treatment options need to be discussed with parents and HCPs should support parents to define and achieve the “good outcome” they want for their child. While some of these children might have life-limiting diseases, they all deserve to be treated with dignity and respect, and HCPs should do all they can to enable them to live the best life possible—however short or long it may be.

## Data Availability

Not applicable.
